# Frequency and Variability of Genomic Rearrangements on *MSH2* in Spanish Lynch Syndrome Families

**DOI:** 10.1371/journal.pone.0072195

**Published:** 2013-09-11

**Authors:** Atocha Romero, Pilar Garre, Olivia Valentin, Julian Sanz, Pedro Pérez-Segura, Patricia Llovet, Eduardo Díaz-Rubio, Miguel de la Hoya, Trinidad Caldés

**Affiliations:** 1 Molecular Oncology Laboratory, Instituto de Investigación Sanitaria San Carlos, Madrid, Spain; 2 Pathology Department, Instituto de Investigación Sanitaria San Carlos, Madrid, Spain; 3 Medical Oncology Department, Instituto de Investigación Sanitaria San Carlos, Madrid, Spain; IFOM, Fondazione Istituto FIRC di Oncologia Molecolare, Italy

## Abstract

Large genomic rearrangements (LGRs) in DNA-mismatch-repair (MMR) genes, particularly among *MSH2* gene, are frequently involved in the etiology of Lynch syndrome (LS). The Multiplex Ligation and Probe Amplification assay (MLPA) is commonly used to identify such alterations. However, in most cases, the MLPA-identified alteration is not characterized at the molecular level, which might be important to identify recurrent alterations and to analyze the molecular mechanisms underlying these mutational events. Probands from a cohort of Lynch Syndrome families were screened for point mutation in MMR genes, subsequently the MLPA assay was used for LGR screening. The identified MLPA alteration was confirmed by cDNA, CGH-microarrays or massive parallel sequencing. In this study, we have delimited the region of 11 LGRs variants on *MSH2* locus. Six of them were fully characterized the breakpoints and 9 of them were considered pathogenic. According to our data, LGR on *MSH2* locus constituted the 10.8% (9 out of 83) of pathogenic germline alterations found in LS. The frequency of colorectal cancer (CRC) and endometrial cancer (EC) in LGR carriers was 55% and 11% respectively. Analysis of the breakpoint sequences revealed that in 3 cases, deletions appeared to originate from Alu-mediated recombination events. In the remaining cases, sequence alignment failed to detect microhomology around the breakpoints. The present study provides knowledge on the molecular characterization of *MSH2* LGRs, which may have important implications in LS diagnosis and Genetic Counseling. In addition, our data suggests that nonhomologous events would be more frequently involved in the etiology of *MSH2* LGRs than expected.

## Introduction

Lynch syndrome (LS) is the most common of the hereditary colon cancer syndromes. It is characterized by a dominantly inherited predisposition to early onset colorectal carcinoma and certain extra colonic tumours, caused by germline mutations in DNA mismatch repair (MMR) genes, most commonly in *MLH1* and *MSH2* [1]–[3].The genetic diagnosis of this inherited predisposition offers an opportunity for intensive targeted clinical surveillance of healthy carriers, which has been proven to reduce significantly cancer morbidity and mortality [4]. On the other hand, the identification of individuals not carrying the family-specific mutation can avoid unnecessary surveillance procedures and alleviate the fear of cancer. Point mutation screening fails to detect pathogenic changes in a considerable percentage of families meeting Amsterdam criteria [4], [5] with large genomic rearrangements (LGRs), particularly among the *MSH2* gene, representing a significant fraction of germline mutations in LS families [6]. Consequently, the screening of LGRs has been incorporated into the routine of most laboratories.

Several methodologies can be used to identify LGRs. Overall, the Multiplex Ligation and Probe Amplification (MLPA) assay might be the most widely used approach for LGR screening in these genes [7]–[9]. However, using MLPA assay LGRs cannot be fully characterized and must be confirmed by other method. The molecular characterization of LGRs is essential to identify recurrent alterations, to identify genotype/phenotype relationships and to analyse the genetic mechanisms underlying these alterations. However, the molecular characterization of LGRs by conventional techniques can be a time consuming and tedious process. High-throughput technologies, such as CGH microarrays or massive parallel sequencing, open the door for feasible LGRs characterization and can potentially overcome such limitations.

The aim of our study was to characterize at the molecular level and to establish the pathogenicity of the LGRs in MSH2 locus found by multiplex ligation-dependent probe amplification (MLPA) assay used to screen our Lynch Syndrome families. To confirm the LGRs found by MLPA, we used CGH microrarrys, cDNA or massive parallel sequencing all changes were confirmed by Sanger sequencing. We were able to delimit the region for 9 variants and to fully characterize the break point for 6 of the 9 variants. The remaining two variants, one was corroborate the MLPA by the study of the cDNA and the other was not possible to characterized.

This is the first long study on LGR in Spanish Lynch Syndrome Families and will contribute to a better diagnostic of this type of families.

## Materials and Methods

### Patients and samples

Suspected Lynch Syndrome (LS) patients were selected through the San Carlos Hospital Cancer Genetic Counseling Unit (Madrid, Spain). Detailed family histories, from at least three generations, and geographic origins were obtained from the proband and participating relatives. Cancer diagnoses and deaths were confirmed by reviewing the medical records, pathology reports or death certificates. Mutation screening of MMR genes were performed previously in 83 index cases from LS families, 48 were Amsterdam I and 35 Amsterdam II criteria [10], [11] and associated with MSI phenotype and loss of MMR protein expression in tumours. The results of the study had been published [12]–[16]. In the present study our cohort; include 15 patients from our 83 LS families that resulted negative for point mutations analysis in MMR genes that were screened for LGR in MMR genes by MLPA.

### Ethics statement

The study was approved by the Hospital Clínico San Carlos Ethics Committee, Madrid, Spain. An informed consent was signed from each participant after appropriate counseling according to the protocols approved by our Institutional Review Boards.

### DNA isolation

Genomic DNA from peripheral blood lymphocytes was extracted using MagNA Pure LC total nucleic acid extraction kit in a MagNA Pure LC instrument (Roche Diagnostics, Penzberg, Germany).

### RNA isolation RT-PCR

Total RNA, from peripheral blood lymphocytes, was extracted using the Qiagen RNeasy Mini Kit (Qiagen Inc., Valencia, CA), following the instructions of the manufacturer. 200 ng of total RNA was used as a template to obtain first-strand cDNA using the SuperScript First-Strand Synthesis System for RT-PCR (Invitrogen, Parsley, UK), following the manufacturer's instructions. The cDNA was further amplified with a primer pairs spanning the deletions (specific sequence primers are available upon request). RT-PCR products were subsequently electrophoresed on agarose gels and sequenced using the ABI-3100 Avant genetic analyzer (Applied Biosystems, USA)

### MLPA

MLPA analysis was performed after comprehensive *MLH1*, *MSH2*, *MSH6*, and *PMS2* mutation scanning (full coding sequence, intron/exon boundaries) considered negative for the presence of germ-line mutations. Screening for *MSH2* LGRs was performed using SALSA MLPA kit P003-B1 and P003-B2 according to instructions provided by the manufacturer's (MRC-Holland, Amsterdam, The Netherlands). All reactions were carried out using 100 ng of DNA. Separation and relative quantification of the peaks was performed in an ABI-3130 genetic analyzer (Applied Biosystems, USA). Variation in peak areas was evaluated by cumulative comparison of samples from the same experiment with GeneScan software (Applied Biosystems, USA). For the assessment of allele dosage, the protocol described by the manufacturer (www.mrc-holland.com) was applied. DNA samples with a dosage value less than 0.7 or greater than 1.2 were confirmed in a second independent reaction.

### CGH microarrays

Samples were hybridized against OncoNIM® Familial Cancer, a 60 k Agilent based custom array-CGH (Nimgenetics; Madrid, Spain). This custom array covers the whole genome with a median spatial resolution of 1 probe per 150 kb, with high density coverage in 20 genes related to familial cancer (100 bp median spatial resolution for these genes, with 1 probe per 50 kb in 5′ and 3′ flanking regions). Hybridizations were performed according to the manufacturer's protocols. A commercially available male DNA sample (Promega, Madison, WI, USA) was used as reference DNA. Microarray data were extracted and visualized using the Feature Extraction Software v10.7 and Agilent Genomic Workbench v.5.0 (Agilent Technologies, Santa Clara, CA) using ADM-2 (set as 10) as aberration detection statistic. Only CNVs with, at least, ten consecutive probes for the 20 selected genes, and five consecutive probes for the whole genome, were analyzed. Genomic build NCBI37 (Hg19) was used for delineating the genomic coordinates of the detected CNVs.

### Long range PCR amplification and massive parallel sequencing

Based on the MLPA data, long-range PCR across the deletion was applied using TAKARA LA PCR kit (TaKaRa Bio Inc., Otsu, Shiga, Japan). Primers used for these analysis and PCR conditions are detailed **table S1**. PCR products were separated on 0.8–1% agarose gels and visualized by ethidium bromide staining. Long-range PCR products containing the expected rearrangement were further purified using Qiaquick PCR purification Kit (Qiagen Inc., Valencia, CA) and quantified using PicoGreen (Molecular Probes, Eugene, OR).

Libraries were synthesized from 500 ng of genomic DNA following the Rapid Library Preparation Method Manual (Roche Applied Science, Mannheim, Germany) and were bar-coded with Rapid Library MID adaptors (Roche 454 life sciences, Mannheim, Germany). The quality of these libraries was analyzed in a Bioanalyser using High Sensitivity DNA Kit (Agilent Technologies Inc., Santa Clara, California, USA). Individual libraries were quantified with qPCR using KAPA Library Quantification kit for Roche 454 Titanium (part KK4802KapaBiosystems Inc., Boston, MA). Based on the individual library concentrations, equimolar pools were made, titrated, and submitted to emulsion-based PCR using GS FLX Titanium LV emPCR kit and GS FLX Titanium emPCR Breaking Kit (Roche Applied Science, Mannheim, Germany), following the manufacturer's instructions. Subsequently, samples were sequenced in GS FLX 454, using a GS FLX Titanium PicoTiterPlate Kit combined with a GS FLX Titanium Sequencing Kit XLR70 (Roche Applied Science, Mannheim, Germany).The average coverage for the captured region ranged from 22.018 reads to 29.036.

Sequencing data was processed using the 454 Sequencing System Software Package v.2.6 (454 Life Sciences Corp, Branford, CT). Reads of high quality were mapped to the reference *MSH2* sequence (Ensembl version: ENSG00000095002.8; genomic region: GRCh37:2:47.605.875 to 47.630.535) using the gsMapper with default parameters. Individual sequences were assembled into contigs by the gsMapper software. Finally, chimeric reads (defined as those which matched to two different regions within the reference) were selected and mapped against the reference sequence. Reads accumulated in two discrete regions were considered the breakpoint of deletions.

### Break point sanger sequencing

Based on CGH-microarrays and massive parallel sequencing results, new PCR were designed using a set of primers that specifically amplified the mutated allele (**Table S1**). PCR products were directly sequenced using the BigDye Terminator v1.1 Cycle Sequencing kit. Sequence analysis was performed on the ABI 3130 genetic analyzer (Applied Biosystems, Foster City, CA, USA). All LGRs are described at the genomic DNA level. The nomenclature for deletions complies with the rules recommended by the Human Genome Variation Society (www.hgvs.org). Genomic break point locations are given in relation to reference sequence for the *MSH2* gene (Ensembl version: ENSG00000095002.8; genomic region: GRCh37:2:47630108-47789450:1; Ensemble release 69).The mentioned *MSH2* reference sequence was submitted to RepeatMasker and was analyzed with default settings.

## Results

### Identification of novel *MSH2* deletions in *MSH2* deficient lynch families

**Figure 1:**
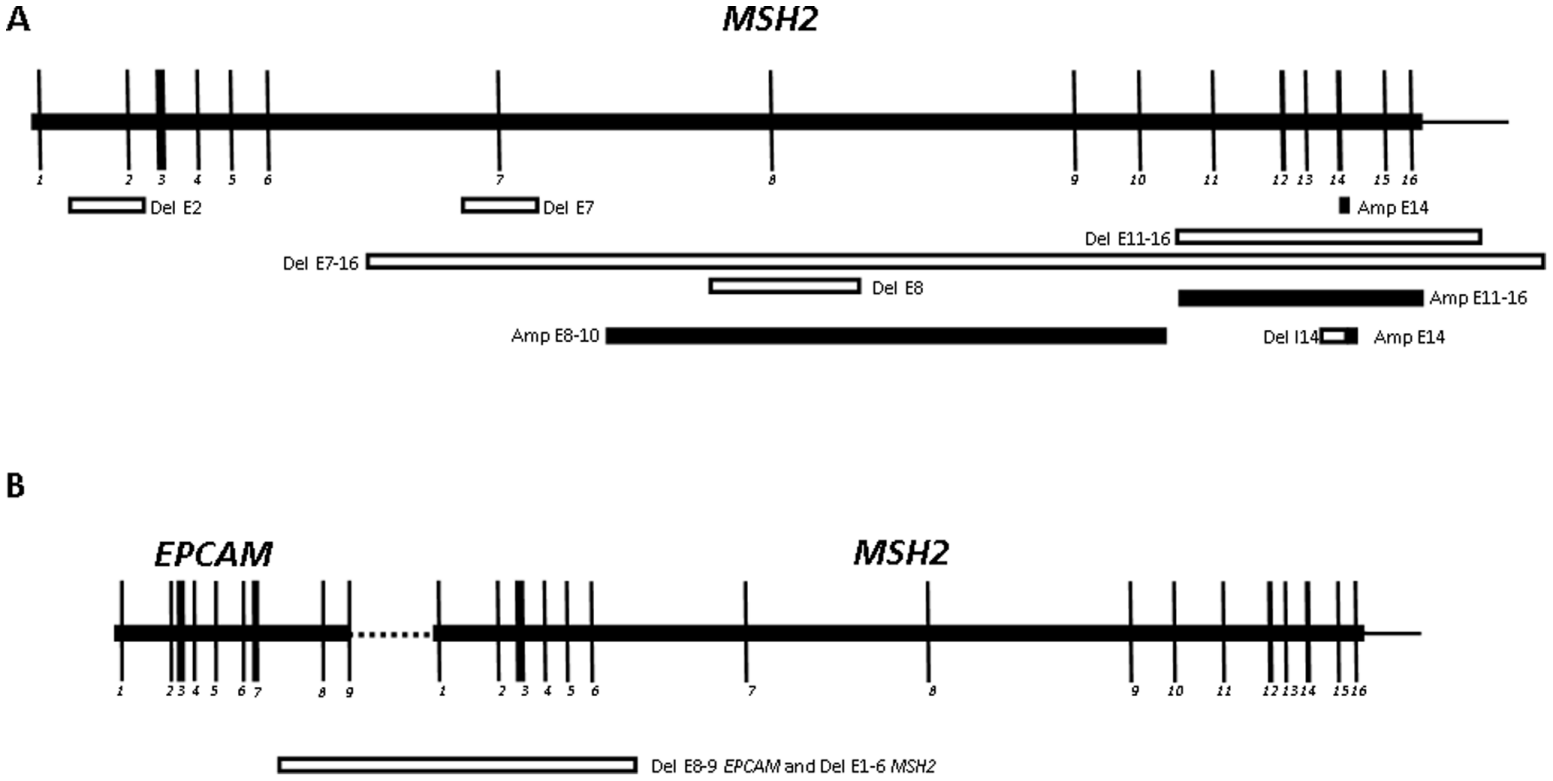
MSH2 LGRs in Lynch syndrome patients. Schematic outline of the genomic region involved in the LGRs, showing 7 deletions (white bars) and 3 amplifications (black bars).

15 Probands that resulted negative for point mutations analysis in MMR genes were submitted to MLPA screening which identified 5 families with putative deletions targeting exon 2, exon 7, exon 8, exons 11–16 and exons 7–16; 3 families with gene duplication that included exon 14, exons 11–16 and exons 8–10 and one family with a deletion targeting exons 8–9 of *EPCAM* gene and exons 1–6 of *MSH2* gene. **Figure 1** outlines the LGRs found in our population.

**Figure 2:**
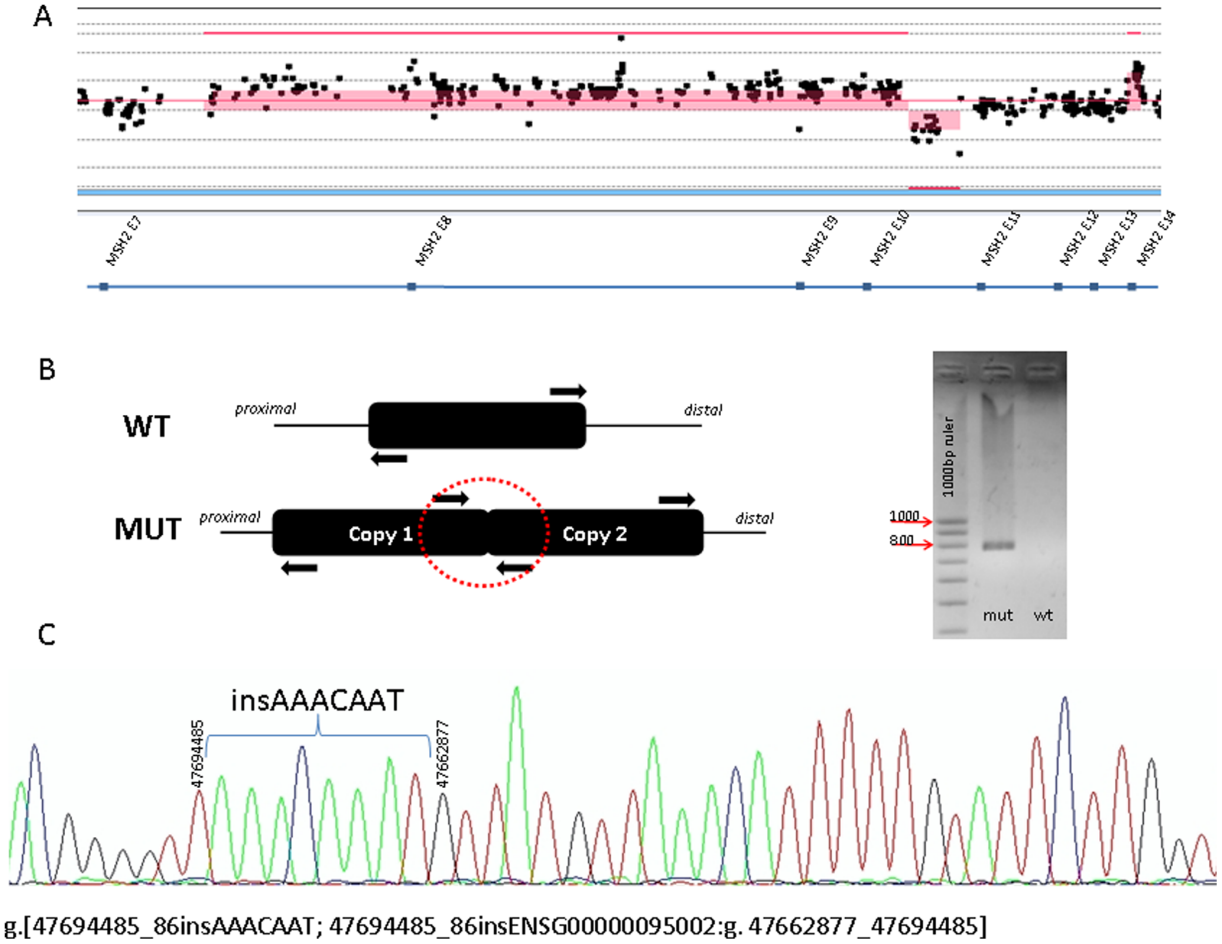
Gene amplification of exons 8–10. **A**) array CGH rearrangement characterization. **B**) Amplification of the junction fragment using the outward facing primers in duplicated head-to-tail interval and electrophoresis gel showing PCR product in a mutation carrier. **C**) Sequence electropherogram of the junction fragment.

We confirmed the MLPA-identified alteration by applying different experimental approaches (**Table 1**). Alleles containing the deletions in exon 7,exons 11–16,and exons 7–16, were further amplified by Long-range PCR using specific primers (**Table S1**) and submitted to massive parallel sequencing. Then, we confirmed the deletion breakpoints by Sanger sequencing using specific primers (**Table S1**). The DNA sample of patient harboring *MSH2* exon 8 deletion was hybridized to a customized array-CGH which provided a prediction of the rearrangement break points. Interesting, this patient also had a deletion in the intronic region of *PTEN* (10q23.31(89652736-89653653)×1). The *PTEN* deletion has been previously reported in healthy individuals with apparently no pathogenic effect. Samples with *MSH2* amplification were also hybridized to the custom array-CGH. The predicted positions flanking the extension of the gene amplification for each sample are detailed in **Table 1**. Remarkable, the proband carrying the gene amplification encompassing exons 8–10 also had a 2.5 Kb deletion in intron 10 (g.47694636-47697106del2471) and a gene amplification involving exon 14 (2p21 (47705272-47705615)×3) (**Figure 2A**). The gene amplification encompassing exons 8–10 was further verified by conventional PCR using the outward facing primers. A PCR product was amplified only in the proband and not in the control (**Figure 2B**). Subsequently, the PCR product was sequenced by Sanger methodology confirming a 31.5 kb duplication preceded by a 7 bp AAACAAT insertion (g.[47694485_86insAAACAAT;47694485_86insENSG00000095002:g.47662877_47694485]) (**Figure 2C**). In addition, the presence of the 2.5 kb deletion was further confirmed by conventional PCR. The analysis of more families members revealed that the 2.5 Kb deletion was in the same allele that the duplication.

**Table 1:** Clinical and molecular characteristics of mutation carriers.

*Family ID*	*Criteria*	*Pedi ID*	*Gender*	*Type of tumor, age at diagnosis*	*Mutation designation*	*MSH2 involved regions*	*Type of alteration*	*Confirmation method*
**21**	AMS I	III:1	M	CRC, 45	c.212-?_366+?del^a^	E2	del	cDNA seq
**65**	AMS I	IV:1	M	CRC, 48	arr 2p21 (47705272-47705637)×3^b^	E14	dup	
		IV:3	M	CRC, 46	arr 2p21 (47705272-47705637)×3^b^	E14	dup	
		IV:6	F	CRC, 39	arr 2p21 (47705272-47705637)×3^b^	E14	dup	arrayCGH
		V:2	M	A, 15	arr 2p21 (47705272-47705637)×3^b^	E14	dup	
		V:4	F	healthy	arr 2p21 (47705272-47705637)×3^b^	E14	dup	
**104**	AMS I	II:2	F	UC, 56; CRC, 65; EC, 70; UC, 76	g.47654696-47659152del4457	E7	del	
		III:1	M	CRC, 38; CRC, 40	g.47654696-47659152del4457	E7	del	cDNA seq
		III:4	M	healthy	g.47654696-47659152del4457	E7	del	MPS
		III:5	M	healthy	g.47654696-47659152del4457	E7	del	DNA seq
**141**	AMS I	III:1	M	healthy	g.47696844-47715548del 18705	E11-16	del	MPS, DNA seq
**499**	AMS II	II:3	M	healthy	g.47649352-47726190del76839	E7-16	del	
		II:4	M	CRC, 68	g.47649352-47726190del76839	E7-16	del	MPS
		III:4	F	healthy	g.47649352-47726190del76839	E7-16	del	DNA seq
		IV:1	F	healthy	g.47649352-47726190del76839	E7-16	del	
**537**	AMS II	III:1	M	CRC, 45; CRC, 59; CRC, 62	g.47672050-47680329del8280,	E8	del	array CGH
		III:6	F	healthy	g.47672050-47680329del8280,	E8	del	cDNA, DNA seq
**639**	AMS II	II:1	M	CRC, 64; UC, 64	arr 2p21 (47696851-47710518)×3^b^	E11-16	dup	
		III:1	M	CRC, 33	arr 2p21 (47696851-47710518)×3^b^	E11-16	dup	
		III:2	M	healthy	arr 2p21 (47696851-47710518)×3^b^	E11-16	dup	array CGH
		III:3	M	healthy	arr 2p21 (47696851-47710518)×3^b^	E11-16	dup	
**481**	AMS I	II:1	M	CRC, 43	2p21 (47661862-47694229)×3^b^, g.47694636-47697106del2471, 2p21 (47705272-47705615)×3	E8-10, I10, E14	dup/del/dup	
		III:1	F	CRC, 32	2p21 (47661862-47694229)mt3^b^, g.47694636-47697106del2471, 2p21 (47705272-47705615)×3	E8-10, I10, E14	dup/del/dup	array CGH
		III:2	M	healthy	2p21 (47661862-47694229)×3^b^, g.47694636-47697106del2471, 2p21 (47705272-47705615)×3	E8-10, I10, E14	dup/del/dup	DNA seq
**677**	AMS II	III:1	F	EC, 39; CRC 41	EPCAM c.859-? _904+?del+MSH2 c.1-?_1076+?del^a^	E1-6	del	
		III:2	F	EC, 45; CRC 46	EPCAM c.859-? _904+?del+MSH2 c.1-?_1076+?del^a^	E1-6	del	
		IV:1	F	healthy	EPCAM c.859-? _904+?del+MSH2 c.1-?_1076+?del^a^	E1-6	del	

a Nomenclature based on mRNA sequence with GenBank Accession Code NM_002354.2.

b Nomenclature according to ISCN (2009).

Abbreviations:Family ID, family identification; Ped ID, pedigree Identification; AMS, Amsterdam criteria; CRC, colorectal cancer; EC, Endometrial cancer; UC, Urothilial cancer; A, Villous Adenoma; MLPA, Multiplex ligation-dependent probe amplification; MPS, Massive Parallel Sequencing.

In total, we precisely localized and sequenced the breakpoints in 6 *MSH2* novel deletions which varied in size from 2471 to 76839. Regarding amplification of exons 11–16 we could not amplify the junction fragment from genomic DNA although we used different primer sets based on CGH array data.

Additionally, the deletions targeting exons 2, 7 and 8 were further characterized at the RNA level. Using specific primers (available upon request), we were able to amplify cDNA from a control and cDNA from deletion carriers, which in all cases yielded a smaller PCR product than the control. Direct sequencing revealed the presence of messengers lacking exons 2, 7 and 8 respectively (**Figure S2**).

Segregation analysis of the LGRs characterized in these families, identified additional LGRs carriers and non carriers. Pedigrees of all families harboring *MSH2* LGRs are available in **Figure S1**.

In six of the MSH2 variants were reported the region in the LOVD and the remaining five were not reported. (**Table S2**). All MSH2 rearrangements breakpoints were not previously reported in the InSiGHT (LOVD) (**Table S2**) and Ensemble data bases and all were established as pathogenic taking into account by the segregation analysis in the families, lost of MSH2 protein expression in the tumors, MSI-H phenotype and a severe phenotype in the family

### Genotype-phenotype correlations

Clinico-pathological features and molecular findings of the LGRs carrier families are detailed in **Table 1**. The mean age at first diagnosis was 42.9 years (range 18–68). At the time of the study 11 *MSH2* LGRs carriers were asymptomatic, two of them aged 74 and 66 (patient III:6 from family 537 and III:3 from family 499 respectively).

According to our results, the frequency of *MSH2* LGRs in Amsterdam I families was 10.4% and 11.4% in Amsterdam II families. On the other hand, based on our data, LGR on *MSH2* locus constitutes the 10.8% (9 out of 83) of pathogenic germline alterations found in LS families in our population and the 20.5% (9 out of 44) of the total alterations found by our group in *MSH2* locus.

The correlation between the type of mutation (punctual or rearrangement) and the phenotype is shown in **Table 2**. There were no differences according to first tumor type, age at first tumor diagnosis or number of tumors developed. According to our data, the 55% of LGR carriers developed CRC compare with 42% in punctual mutation carries however this difference did not reach statistical significance. On the other hand, endometrial cancer (EC) was diagnosed in 11% of LGR carriers and in 14% of punctual mutation carriers.

**Table 2:** Genotype-phenotype correlation in MSH2 mutation carriers.

	*Punctual mutation*	*LGR*	*P*
*Number of affected*	54	16	
*Number of healthy*	53	11	0.547
*Number of tumors*			
*1 tumor*	36	10	
*>1 tumor*	18	6	0.993
*Number of individuals developing… .*			
*Colorectal cancer*	45	15	0.278
*Endometrial cancer*	15	3	0.936
*Age at diagnosis first cancer (average)*	42	43	0.823

### LGRs mechanisms of origin

In order to elucidate the molecular mechanisms underlying the origin of LGRs in *MSH2* gene we analyzed the entire sequence of *MSH2* gene (genomic region: GRCh37:2:47630108-47789450:1). Analysis with default settings identified 168 SINEs, 39 LINEs, 33 LTRs, and 29 DNA elements. Together, these repeat elements comprise 47.46% of the whole sequence, indicating a relatively high density of repetitive *Alu* elements within this region. Simple inspection permitted us to notice that breakpoints, in some cases, were located at interspersed repeated elements. Three *MSH2* deletion breakpoints characterized in this study were located within *Alu* repeats (**Table 3**).The two recombined *Alu* elements were always directed in the same orientation (**Figure S3**). Sequence alignments of the proximal and distal *Alu* sequences revealed the presence of stretches with microhomology at the breakpoint, ranging in size from 15 to 48 bp (**Table 3**) (**Figure S3**), indicating that, in these cases deletions might have arisen by *Alu-Alu* mediated nonallelic homologous recombination (NAHR). However, this mechanism does not explain g.47672050-47680329del8280, and g.47694636-47697106del2471 rearrangements in which nonhomologous end-joining (NHEJ) may serve as a better explanation for the origin of the deletions. In these patients, sequence alignment of the regions surrounding the breakpoints discarded both non-allelic homologous recombination and micro-homology mediated events, despite the fact that in case of g.47694636-47697106del2471 5′ and 3′ breakpoints were embedded in interspersed repeated sequences. Similarly, in case of exon 8 deletion (g.47672050-47680329del8280) the sequence surrounding the breakpoint at 5′ corresponded to *AluSx*. In the same way, alignment analysis of exons 8–10 amplification junction fragment failed to detected stretches of homology at the breakpoints, therefore discarding homologous recombination as the mechanisms of origin for such alteration although the breakpoint at 3′ was embedded in a *AluSx* sequence.

**Table 3:** Breakpoint mapping and evaluation of genomic features at the breakpoints.

Family ID	Mutation designation	Exons involved	Microhomology	Repetitive element 5′	Repetitive element 3′	Proposed mechanism
**HC-104**	g.47654696-47659152del4457	E7	24	Alu Y	Alu Sp	NAHR
**HC-141**	g.47696844-47715548del 18705	E11-16	48	Alu Y	Alu Y	NAHR
**HC-499**	g.47649352-47726190del76839	E7-16	15	Alu Jb	Alu Sz	NAHR
**HC-537**	g.47672050-47680329del 8280	E8	-	Alu Sx	-	NHE
**HC-481**	g.47694636-47697106del2471	I10	-	-	-	NHE
**HC-481**	47694485_86insENSG00000095002:g.47662878_47694485	E8-10	-	-	AluSx	NHE

## Discussion

In this study, we report the characterization at the molecular level of 9 novel structural alterations on the *MSH2* locus in patients with LS based on clinical and immunohistochemicals findings and that resulted negative for point mutations analysis in MMR genes. According to our results, the prevalence of *MSH2* LGRs in Amsterdam I and II families was 10.4% and 11.4% respectively. In our study *MSH2* deletions constituted 10.8% of pathogenic germline alterations found in LS families, indicating that LGRs account for non negligible proportion of *MSH2* mutations, which is in accordance with previously LGRs rates reported from similar series [5], [6], [17]–[19].

The spectrum of tumors developed in carriers, of Spanish families harboring *MSH2* LGR, were mostly CRC. The frequency of CRC in LGRs carriers was higher than in point mutation carriers while the opposite was observed for EC. However, as others before, we failed to demonstrate phenotypic significant differences of families carrying the detected rearrangements and families harboring other types of mutations [8], [17].

Six of the detected rearrangements were deletions. The deletion .47694636-47697106del2471 has been found in family 481 and affects intron 10. We didn't consider it as pathogenic because it has been found in co-ocurrence with the pathogenic duplication of exons 8–10. In the remaining cases, the rearrangement creates a premature stop codon that would produce a putative truncated protein or an in-frame deletion, affecting important functional domains of the protein. Four rearrangements consist of amplifications. In case of the *MSH2* amplification of exons 8–10, we were able to sequence the junction fragment, therefore demonstrating the pathogenic significance of this alteration. Amplification of exon 14 and ex 11–16 was not possible to localize the exact breakpoint. We didn't considered the amplification of the *PTEN* because has been reported in healthy individuals with no pathogenic effect.

LGRs can be generated through different genetic mechanisms such as NAHR, micro-homology mediated events, involving very short homologous sequences, or homology-independent processes such as classical NHEJ [20]–[22]. It is well established that there is a relatively high local density of repetitive Alu elements throughout *MSH2* locus [23]–[26] increasing the chance of Alu-mediated recombination, which might explain the wide variety of deletions within this region. In our study, we have found that in some cases LGRs breakpoints fall within repetitive sequences. Specifically, we have found Alu elements to be involved in 3 LGRs, indicating that in these cases meiotic NAHR could be the most likely underlying mechanism. In case of g.47672050-47680329del8280 and g.47694636-47697106del2471 rearrangements, the sequences surrounding the breakpoints did not contain enough homology despite the fact that breakpoints were embedded within repeated elements, suggesting that, in these cases, LGRs might be generated by a non-homologous mechanism as NHEJ. The same can be argued for exons 8–10 amplification. Therefore, indicating that location of breakpoints at Alu sequences is not a proxy for NAHR. Alu-mediated NAHR has been proposed as the most frequent mechanism underlying *MSH2* LGRs [24], while homology-independent processes are considered to be exceptional. Nonetheless, homology-independent processes have been involved in LGRs that cause predisposition to colon cancer before [26], [27]. According to our data, non-homologous mechanisms in LGRs generation could have been underestimated and would be more frequent than previously expected despite the relatively high density of Alu repeat elements within *MSH2* locus. Similar conclusions have been reached in BRCA2 LGRs carriers [28]. Analysis of lagers cohorts of LGRs carriers would be clearly warranted in order to clarify this issue.

High throughput technologies allow nowadays accurately detecting and characterizing these classes of mutations, diminishing substantially the time of analysis. In our study we were able to confirm the previously detected MLPA alteration by CGH-microarrays and massive parallel sequencing. Guidelines for application of high throughput technologies to genetic diagnostic have been developed [29]. In our view, we believe that standard Sanger sequencing should be applied to verify positive results as well as it provides the basis for a simplified test for high risk relatives.

In conclusion, we have reported 9 novel pathogenic mutations causing LS. Our data suggests that LGRs may explain a significant proportion of point mutation negative families with MMR protein loss and MSI-H phenotype in tumor tissue. Moreover, our data suggests that non-homologous mechanisms would be more frequently involved in the etiology of *MSH2* LGRs than estimated.

The incorporation of novel high throughput technologies to routine analysis will enable the characterization of this class of mutation more easily. The identification of these variants is important for diagnosis, genetic counseling and management of the patients and families with Lynch syndrome.

## Supporting Information

Figure S1 **Pedigrees of families harboring LGRs in MSH2.**
(PPTX)

Figure S2 **Study at cDNA level of the three patients carrying deletions of exons 2, 7 and 8.**
(TIF)

Figure S3 **Alignments of** ***MSH2*** **deleted allele with 5′ and 3′ sequences.** The boxed sequence indicates the microhomology at the breakpoint region.
(TIF)

Table S1 **Primers used in the current study.**
(DOC)

Table S2 **MSH2 variants in the LOVD database.**
(DOC)
