# Supplementary material for: Frequency and Variability of Genomic Rearrangements on MSH2 in Spanish Lynch Syndrome Families
Source: PLoS One. 2013 Sep 11;8(9):e72195. doi: 10.1371/journal.pone.0072195 (PMC3770653; doi:10.1371/journal.pone.0072195)
Supplement: Figure S1 — Pedigrees of families harboring LGRs in MSH2. (PPTX) [file pone.0072195.s001.pptx]

## Slide 1
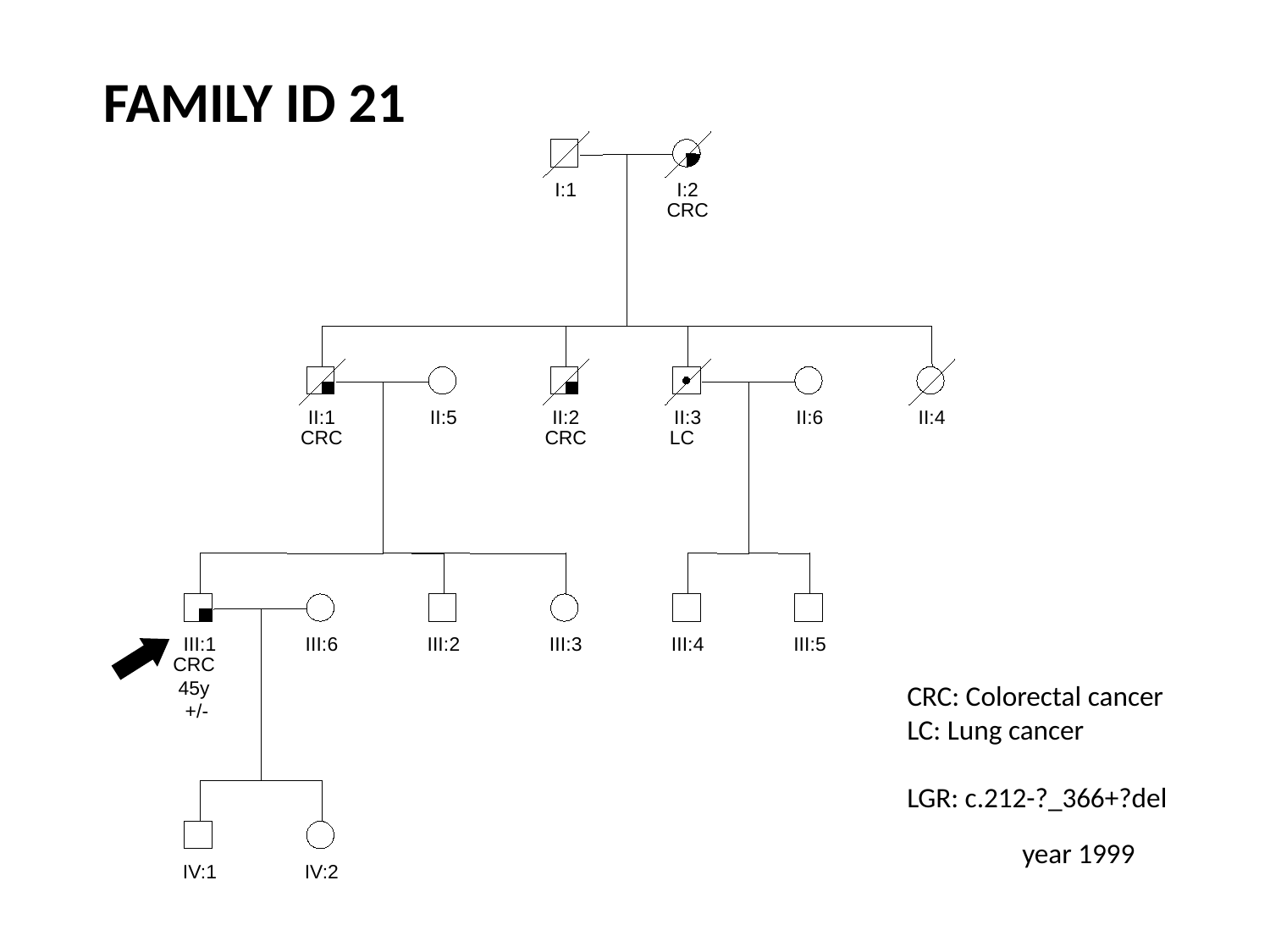

FAMILY ID 21
I:1
I:2
CRC
II:1
II:5
II:2
II:3
II:6
II:4
CRC
CRC
LC
III:1
III:6
III:2
III:3
III:4
III:5
CRC
45y
 +/-
IV:1
IV:2
CRC: Colorectal cancer
LC: Lung cancer
LGR: c.212-?_366+?del
year 1999

## Slide 2
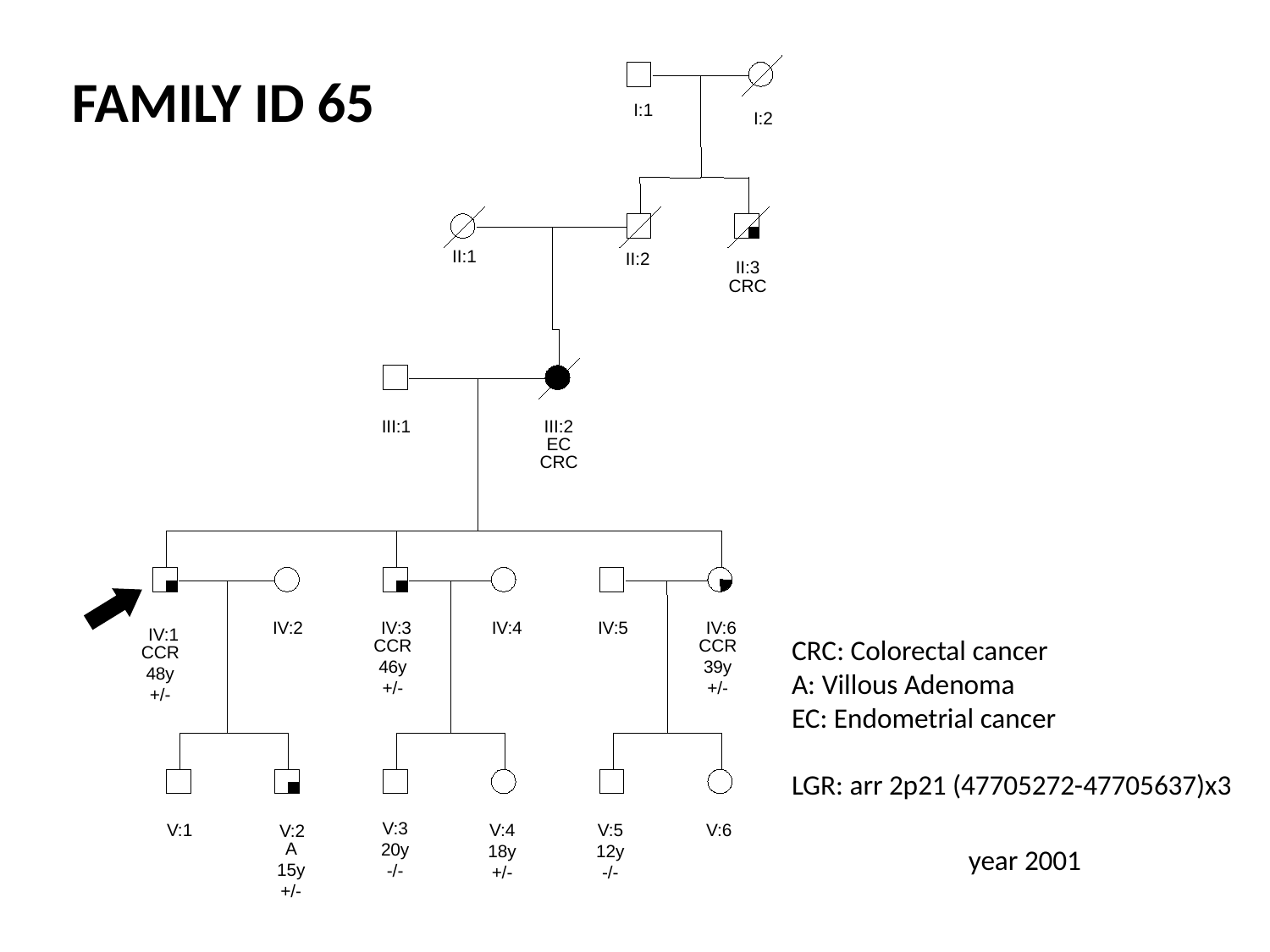

I:1
I:2
II:1
II:2
II:3
CRC
III:1
III:2
EC
CRC
IV:4
IV:2
IV:3
IV:5
IV:6
IV:1
CCR
46y
+/-
CCR
39y
+/-
CCR
48y
+/-
V:3
20y
-/-
V:1
V:4
18y
+/-
V:5
12y
-/-
V:6
V:2
A
15y
+/-
FAMILY ID 65
CRC: Colorectal cancer
A: Villous Adenoma
EC: Endometrial cancer
LGR: arr 2p21 (47705272-47705637)x3
year 2001

## Slide 3
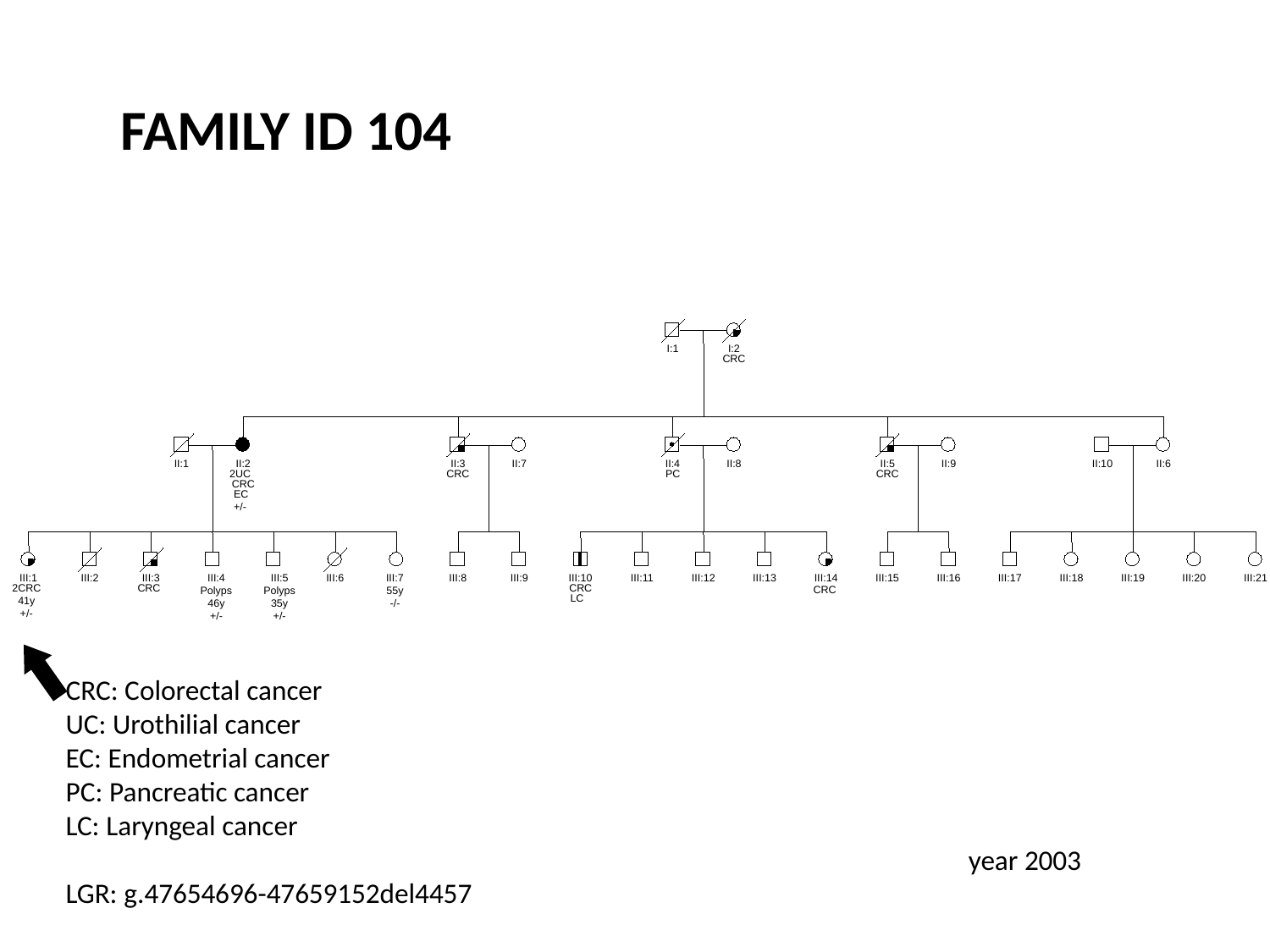

FAMILY ID 104
I:1
I:2
CRC
II:1
II:2
II:3
II:7
II:4
II:8
II:5
II:9
II:10
II:6
2UC
CRC
PC
CRC
CRC
EC
+/-
III:1
III:2
III:3
III:4
Polyps
46y
+/-
III:5
Polyps
35y
+/-
III:6
III:7
55y
-/-
III:8
III:9
III:10
III:11
III:12
III:13
III:14
III:15
III:16
III:17
III:18
III:19
III:20
III:21
2CRC
41y
+/-
CRC
CRC
LC
CRC
CRC: Colorectal cancer
UC: Urothilial cancer
EC: Endometrial cancer
PC: Pancreatic cancer
LC: Laryngeal cancer
LGR: g.47654696-47659152del4457
year 2003

## Slide 4
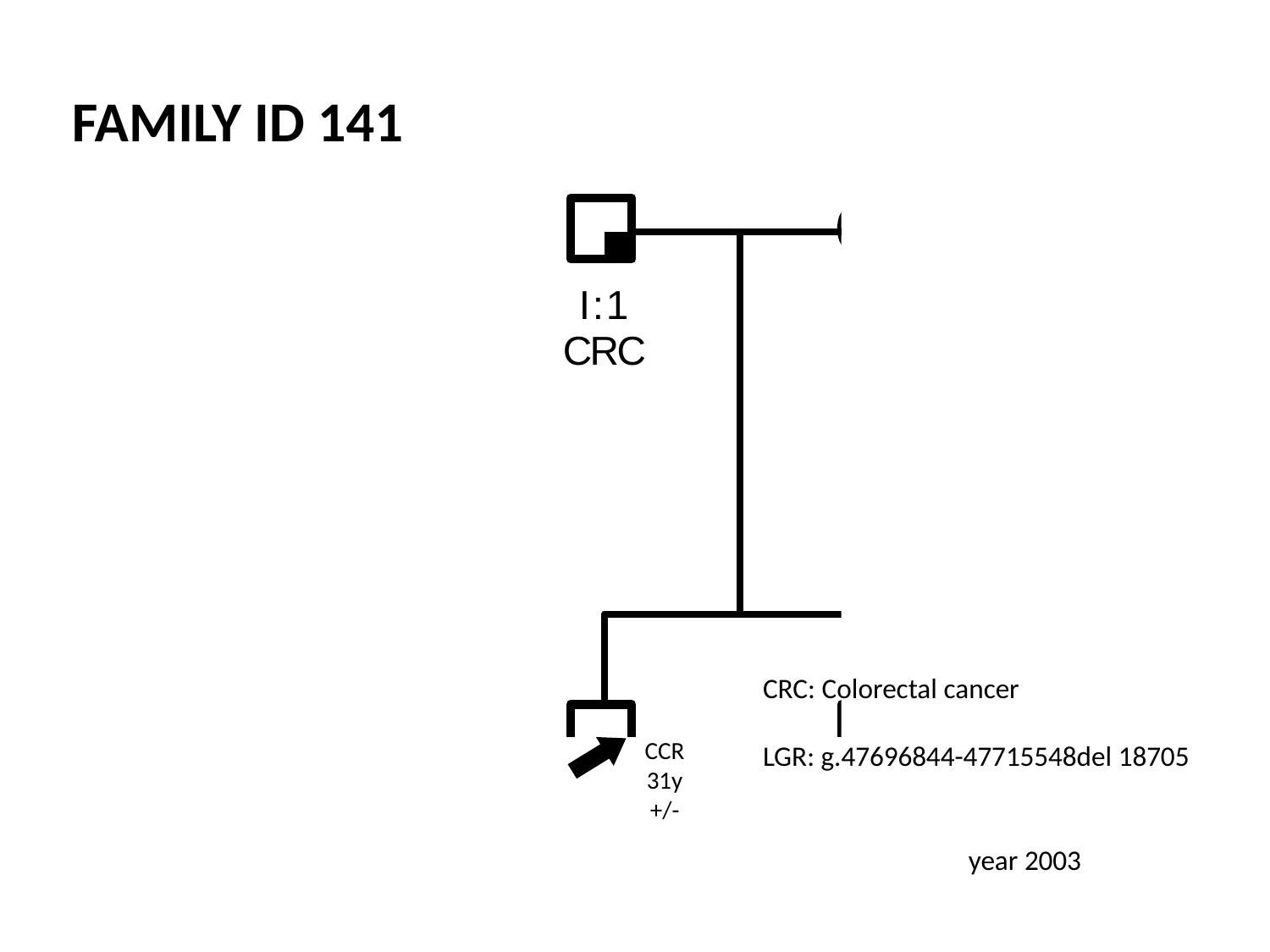

FAMILY ID 141
CRC: Colorectal cancer
LGR: g.47696844-47715548del 18705
CCR
31y
+/-
year 2003

## Slide 5
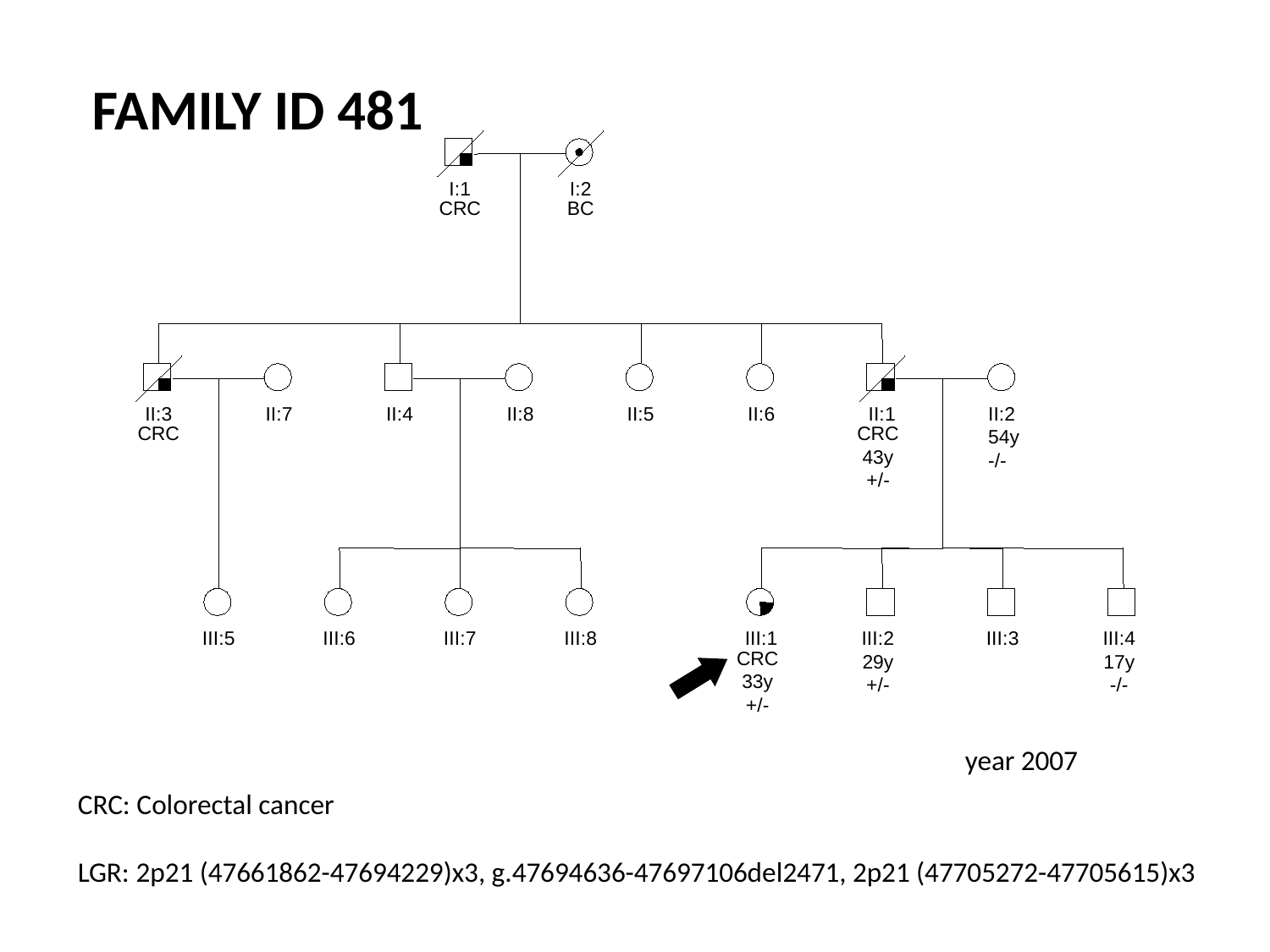

FAMILY ID 481
I:1
I:2
CRC
BC
II:3
II:7
II:4
II:8
II:5
II:6
II:1
II:2
54y
-/-
CRC
CRC
43y
+/-
III:5
III:6
III:7
III:8
III:1
III:2
29y
+/-
III:3
III:4
17y
-/-
CRC
33y
+/-
year 2007
CRC: Colorectal cancer
LGR: 2p21 (47661862-47694229)x3, g.47694636-47697106del2471, 2p21 (47705272-47705615)x3

## Slide 6
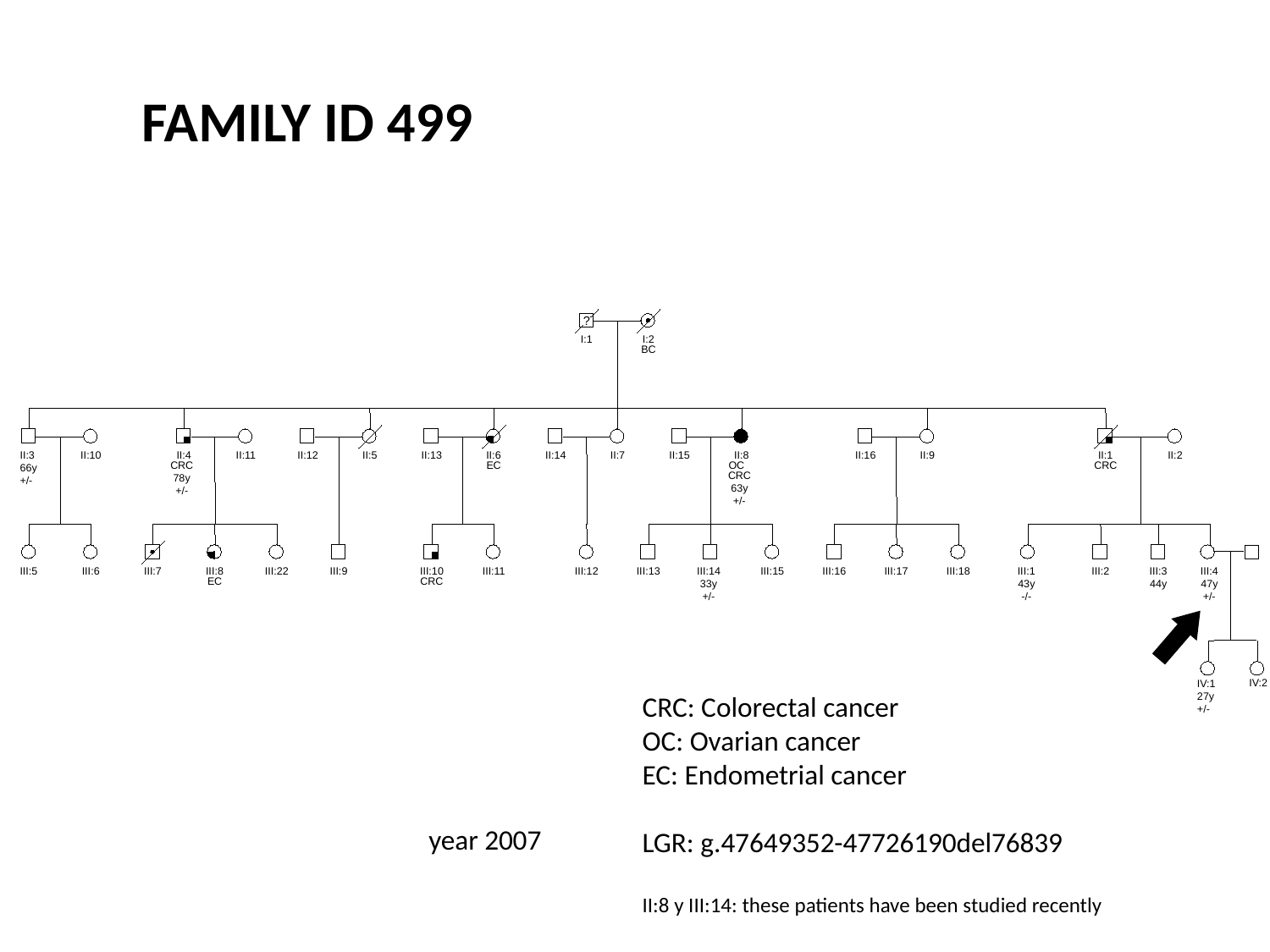

FAMILY ID 499
?
I:1
I:2
BC
II:3
66y
+/-
II:10
II:4
II:11
II:12
II:5
II:13
II:6
II:14
II:7
II:15
II:8
II:16
II:9
II:1
II:2
CRC
78y
+/-
EC
OC
CRC
CRC
63y
+/-
III:5
III:6
III:7
III:8
III:22
III:9
III:10
III:11
III:12
III:13
III:14
33y
+/-
III:15
III:16
III:17
III:18
III:1
43y
-/-
III:2
III:3
44y
III:4
47y
+/-
EC
CRC
IV:2
IV:1
27y
+/-
CRC: Colorectal cancer
OC: Ovarian cancer
EC: Endometrial cancer
LGR: g.47649352-47726190del76839
II:8 y III:14: these patients have been studied recently
year 2007

## Slide 7
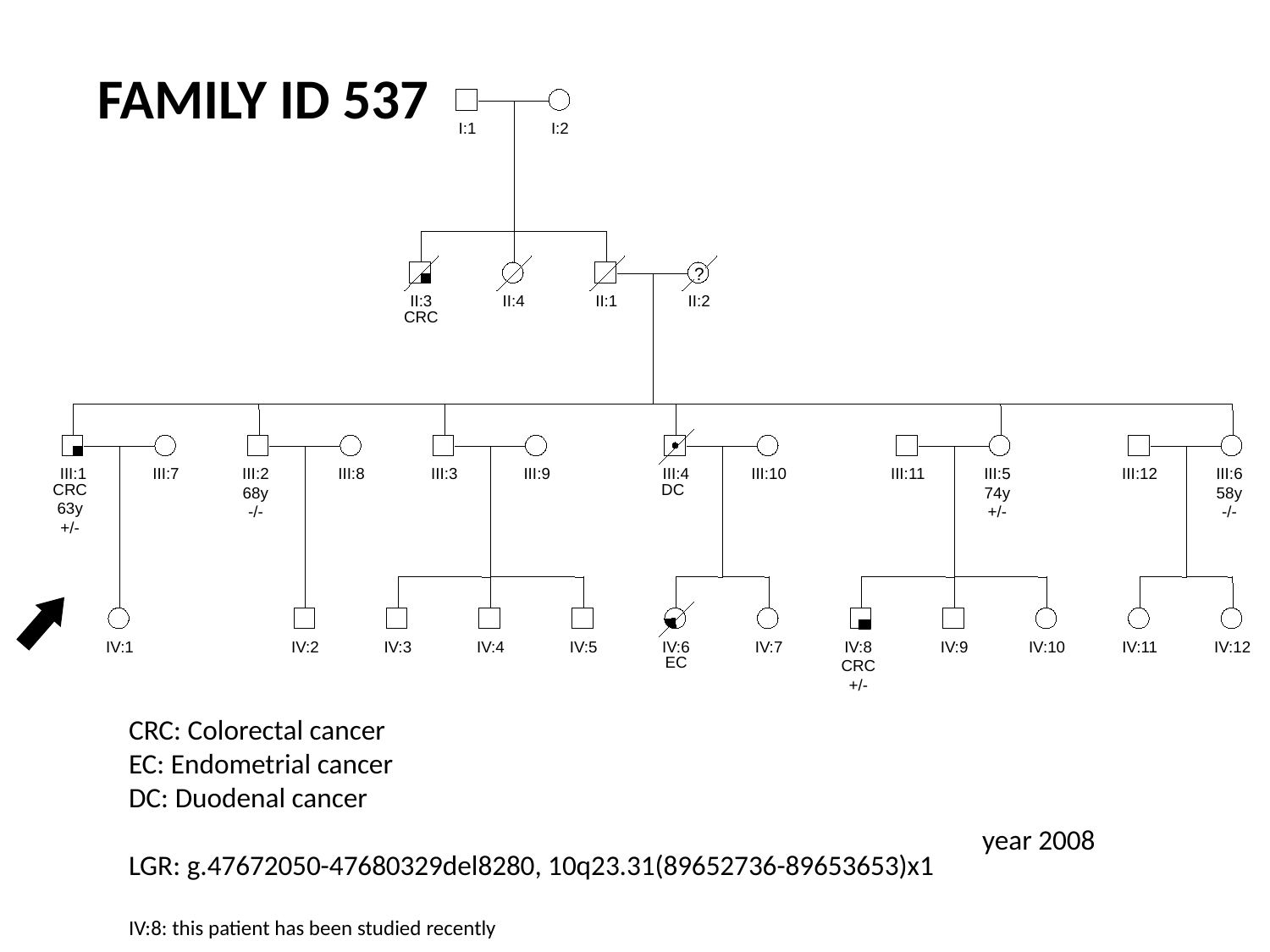

I:1
I:2
?
II:3
II:4
II:1
II:2
CRC
III:1
III:7
III:2
68y
-/-
III:8
III:3
III:9
III:4
III:10
III:11
III:5
74y
+/-
III:12
III:6
58y
-/-
CRC
63y
+/-
DC
IV:1
IV:2
IV:3
IV:4
IV:5
IV:6
IV:7
IV:8
CRC
+/-
IV:9
IV:10
IV:11
IV:12
EC
FAMILY ID 537
CRC: Colorectal cancer
EC: Endometrial cancer
DC: Duodenal cancer
LGR: g.47672050-47680329del8280, 10q23.31(89652736-89653653)x1
IV:8: this patient has been studied recently
year 2008

## Slide 8
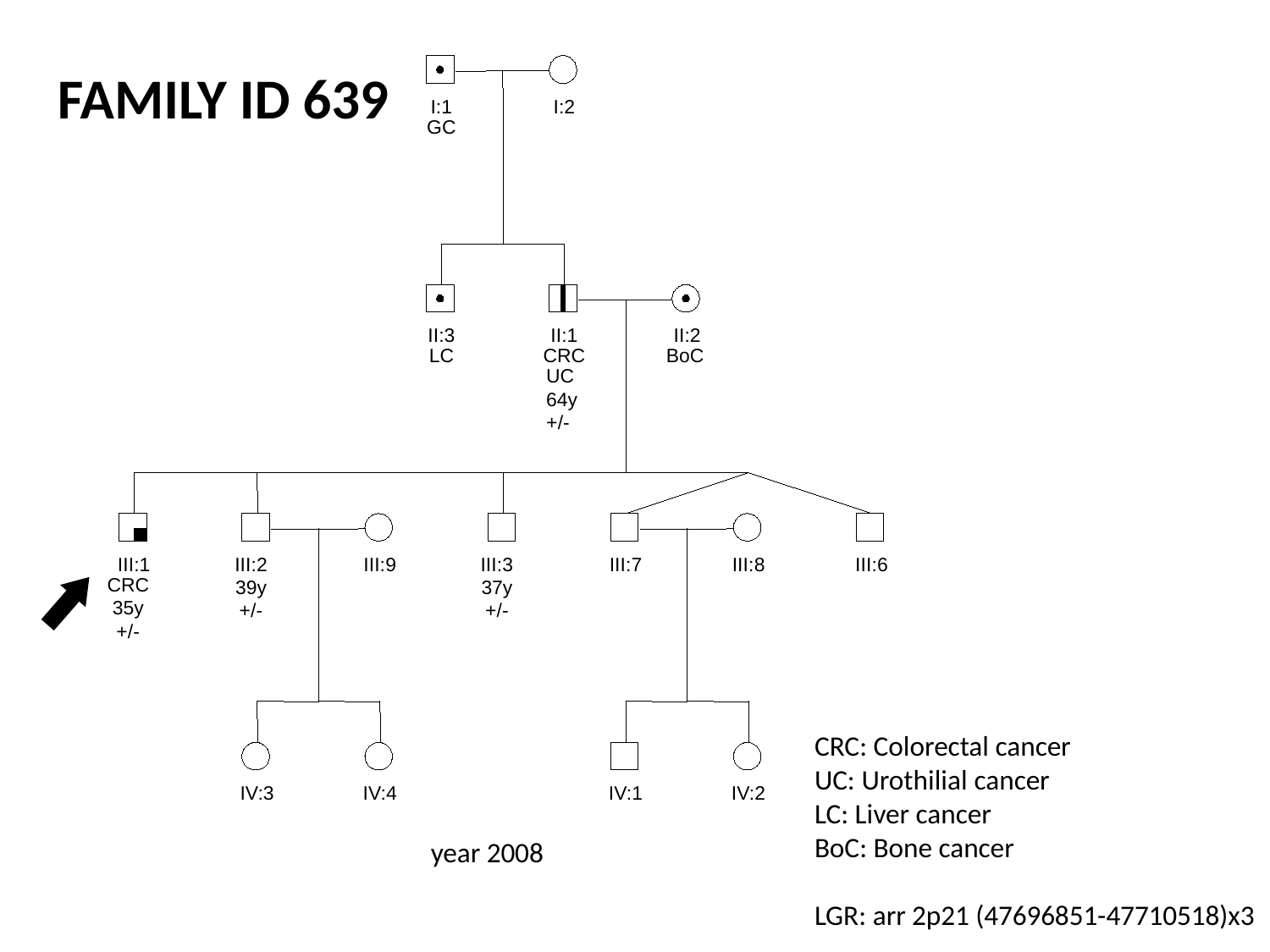

I:1
I:2
GC
II:3
II:1
II:2
LC
CRC
BoC
UC
64y
+/-
III:1
III:2
39y
+/-
III:9
III:3
37y
+/-
III:7
III:8
III:6
CRC
35y
+/-
IV:3
IV:4
IV:1
IV:2
FAMILY ID 639
CRC: Colorectal cancer
UC: Urothilial cancer
LC: Liver cancer
BoC: Bone cancer
LGR: arr 2p21 (47696851-47710518)x3
year 2008

## Slide 9
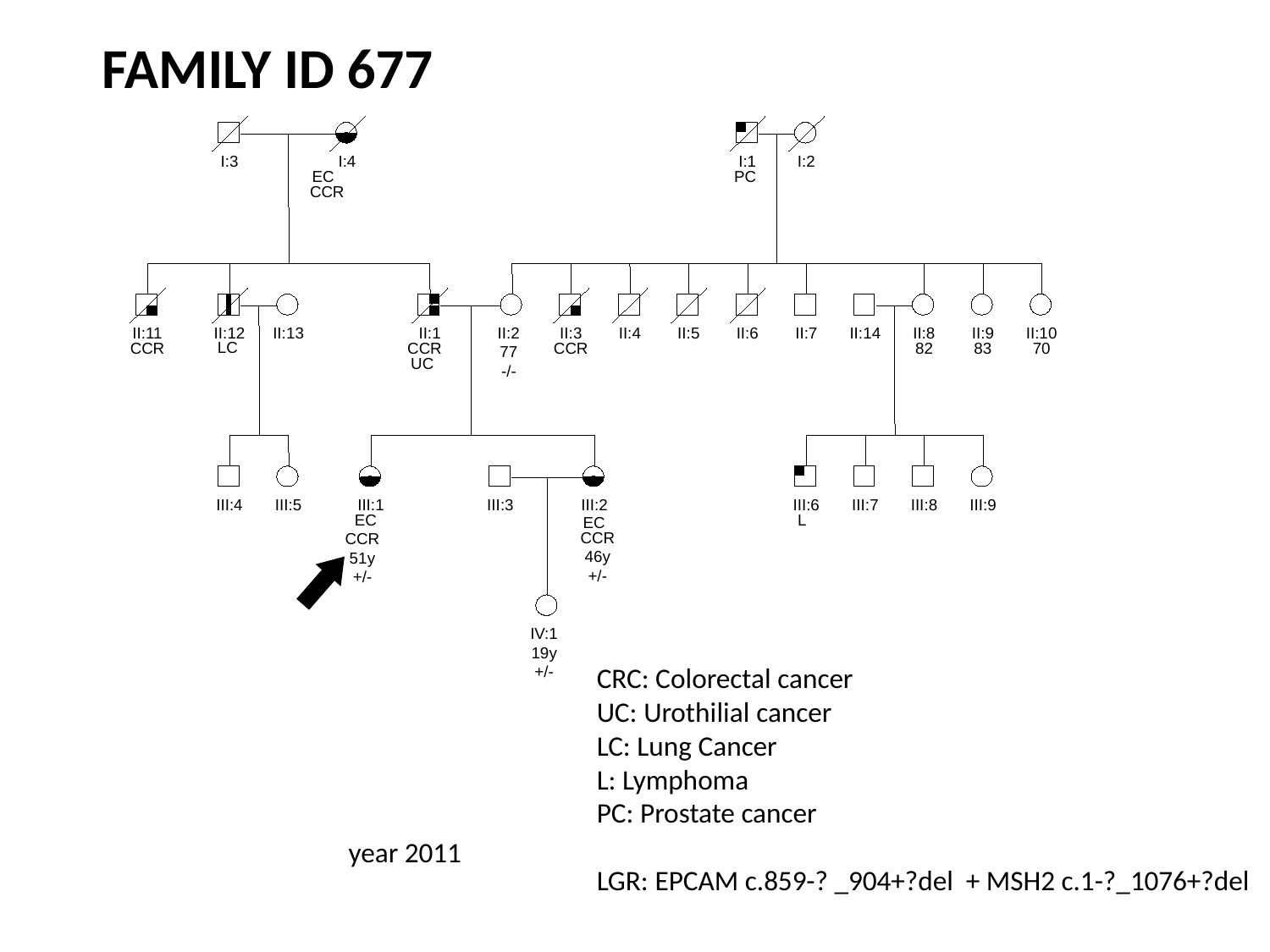

FAMILY ID 677
I:3
I:4
I:1
I:2
EC
PC
CCR
II:11
II:12
II:13
II:1
II:2
77
-/-
II:3
II:4
II:5
II:6
II:7
II:14
II:8
II:9
II:10
LC
CCR
CCR
CCR
82
83
70
UC
III:4
III:5
III:1
III:3
III:2
III:6
III:7
III:8
III:9
EC
L
CCR
51y
+/-
IV:1
19y
+/-
EC
CCR
46y
+/-
CRC: Colorectal cancer
UC: Urothilial cancer
LC: Lung Cancer
L: Lymphoma
PC: Prostate cancer
LGR: EPCAM c.859-? _904+?del + MSH2 c.1-?_1076+?del
year 2011
