# Supplementary figures and images for: Frequency and Variability of Genomic Rearrangements on MSH2 in Spanish Lynch Syndrome Families
Source: PLoS One. 2013 Sep 11;8(9):e72195. doi: 10.1371/journal.pone.0072195 (PMC3770653; doi:10.1371/journal.pone.0072195)

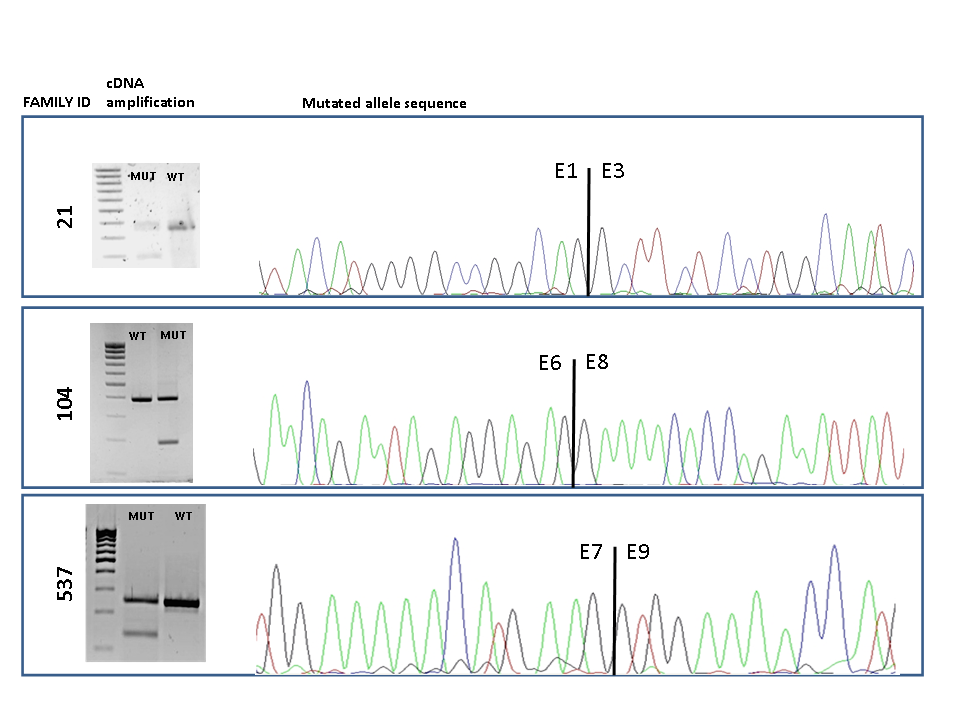

Supplement: Figure S2 — Study at cDNA level of the three patients carrying deletions of exons 2, 7 and 8. (TIF) [file pone.0072195.s002.tif]

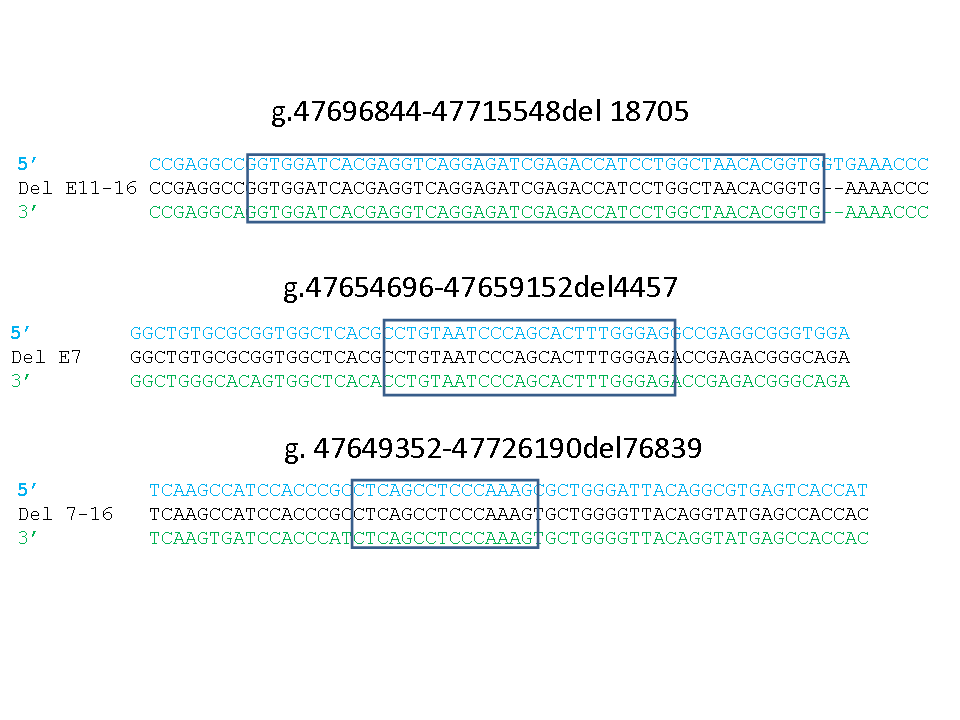

Supplement: Figure S3 — Alignments of MSH2 deleted allele with 5′ and 3′ sequences. The boxed sequence indicates the microhomology at the breakpoint region. (TIF) [file pone.0072195.s003.tif]
