# Supplementary material for: Frequency and Variability of Genomic Rearrangements on MSH2 in Spanish Lynch Syndrome Families
Source: PLoS One. 2013 Sep 11;8(9):e72195. doi: 10.1371/journal.pone.0072195 (PMC3770653; doi:10.1371/journal.pone.0072195)
Supplement: Table S1 — Primers used in the current study. (DOC) [file pone.0072195.s004.doc]

Table 1 S: Sequences of the primers used for LR-PCR and Sequencing

| **Application** | Gene | Exons involved | **Primer name** | **Sequence (5'-3')** | **Coordinates** | **Tm (ºC)** |
| --- | --- | --- | --- | --- | --- | --- |
| **Long range PCR** | EPCAM | 8-9 | EPCAMI7FW | GTTGCATGGCTGTCTTATTTCC | 47605875-47605896 | 58 |
|  |  |  | EPCAM3'REV | TTATTGCAAATCGAGAAGTACG | 47623927-47623948 | 55 |
|  | MSH2 | 7 | MSH2I6.3FW | GTGCATAATAATTGCATGGGA | 47653803-47653823 | 56 |
|  |  |  | MSH2I7REV | ACAAGGGGGATACATGGCTG | 47659451-47659470 | 59 |
|  | MSH2 | 11-16 | MSH210FW | GGTAGTAGGTATTTATGGAATAC | 47693748-47693770 | 49 |
|  |  |  | MSH2.3.25REV | CATACCACTTGCTTTGACAC | 47715996-47716015 | 53 |
|  | MSH2 | 7-16 | MSH2I6FW | CAACTCAGTGAGACGCCATC | 47644333 -47644352 | 58 |
|  |  |  | MSH216KBREV | CTCCTGCAGTGCCTTAGCA | 47726524-47726542 | 59 |
| **Sequencing** | EPCAM | 8-9 | EPCAMS | TTGCTTGTAAAGGTGAATCTGG | 47609524-47609545 | 57 |
|  |  |  | EPCAM3'REV | TTATTGCAAATCGAGAAGTACG | 47623927-47623948 | 55 |
|  | MSH2 | 7 | MSH2I6.2FW | TGTAAATGCTGGTGATGTCAGT | 47654610-47654631 | 57 |
|  |  |  | MSH2I7REV | ACAAGGGGGATACATGGCTG | 47659451-47659470 | 59 |
|  | MSH2 | 8 | MSH2I7FW | CCTCCTGAGGATGTTTGACATT | 47667410-47667431 | 60 |
|  |  |  | MSH2I8.2REV | CCTTCCCAGGAGTGCTCAGAC | 47680520 -47680540 | 63 |
|  | MSH2 | 8-10 | MSH210FW | GGTAGTAGGTATTTATGGAATAC | 47693748 -47693770 | 50 |
|  |  |  | MSH210NESTEDFW | GGAGTCTCGCTCTGTTGC | 47694313-47694330 | 58 |
|  |  |  | MSH2I76000REV | GAAAACCAAGAATACAAAAATGTTT | 47662985- 47663009 | 53 |
|  | MSH2 | I-10 | MSH2I10FW | TTTTTTCCCCCTGAGACAGA | 47694589-47694607 | 55 |
|  |  |  | MSH211REV | CCAGGTGACATTCAGAAC | 47698225 -47698242 | 50 |
|  | MSH2 | 11-16 | MSH2I10FW | TTTTTTCCCCCTGAGACAGA | 47694589-47694607 | 55 |
|  |  |  | MSH2.3.25REV | CATACCACTTGCTTTGACAC | 47715996-47716015 | 53 |
|  | MSH2 | 7-16 | MSH2I6.10FW | TGGTACCTCATGCTTCCTAGTT | 47646623-47646644 | 58 |
|  |  |  | MSH216KBREV | CTCCTGCAGTGCCTTAGCA | 47726524-47726542 | 59 |
