# Supplementary material for: Frequency and Variability of Genomic Rearrangements on MSH2 in Spanish Lynch Syndrome Families
Source: PLoS One. 2013 Sep 11;8(9):e72195. doi: 10.1371/journal.pone.0072195 (PMC3770653; doi:10.1371/journal.pone.0072195)
Supplement: Table S2 — MSH2 variants in the LOVD database. (DOC) [file pone.0072195.s005.doc]

Table S2: MSH2 variants in the LOVD database

| **MUTATION** | **Exons involved** | **LOVD reported region** | **LOVD reported**  **breakpoint** |
| --- | --- | --- | --- |
| c.212-?_666+del | E2 | c.212-?_666+del |  |
| arr2p21(47705272-47705637) | E14 | not reported |  |
| g.47654696-47659152del4457 | E7 | c.1077-?_1276+?del | no |
| g.47696844-47715548del18705 | E11-16 | c.1662-?_2805+?del | no |
| g.47649352-47726190DEL76839 | E7-16 | c.1076-?_2805+?del | no |
| g.47672050-47680329del8280 | E8 | c.1277-?_1386+?del | no |
| arr2p21(47696851-47710518)x3 | E11-16 | c.1662-?_2805+?dup |  |
| 47694485_86insENSG00000095002:g.47662878_47694485 | E8-10 | not reported | no |
| g.47694636-47697106del2471 | I10 | not reported | no |
| Arr2p21(47705272-47705615) | E14 | not reported |  |
| EPCAMc.859-?_904+?del+MSH2c.1-?_1076+?del | E1-6 + 8+9 EPCAM | not reported |  |

Arr: CGH Array; g: genomic; c.:cDNA
